# Bilateral Carotid Body Paraganglioma: A Case Report and Review of Management Strategies

**DOI:** 10.1055/a-2654-2376

**Published:** 2025-07-24

**Authors:** Louissa S. R. Cidral, Luiz D. Penzo, Kristel B. Merida, Mayara S. Marques, Ana C. Andrade, Afonso H. Aragão, Leonardo G. Ruschel

**Affiliations:** 1 Neurology and Neurosurgery Department, NeuroDOC, Curitiba, Brazil; 2 Neurology and Neurosurgery Department, Hospital Santa Casa de Curitiba, Curitiba, Paraná, Brazil

**Keywords:** paraganglioma, carotid body tumor, bilateral neck mass, syncope, angiography

## Abstract

Bilateral paragangliomas are rare neuroendocrine tumors stemming from the paraganglia along the autonomic nervous system. This case report presents a case of a 49-year-old woman with a year history of bilateral neck masses and recurrent syncopal episodes. Diagnostic imaging revealed bilateral, hypervascular carotid body tumors. This case underlines the importance of recognizing paragangliomas as a differential diagnosis in patients with neck masses and highlights the role of advanced imaging techniques in diagnosis and management.

## Introduction

Paragangliomas, including carotid body tumors (CBTs), are rare, predominantly benign neuroendocrine neoplasms originating from paraganglia, clusters of cells associated with the autonomic nervous system. Bilateral CBTs are especially uncommon, representing less than 5% of cases. 1 These tumors can display diverse clinical manifestations, one of the most common being neck masses. 2 This report documents a patient with bilateral carotid body paragangliomas, detailing the clinical presentation, diagnostic evaluation, and therapeutic strategy that was pursued.

## Case Report

A 49-year-old woman presented with an initial complaint of bilateral neck masses persisting for 1 year, accompanied by recurrent syncopal episodes. The patient denied pain, dysphagia, or changes in voice. Physical examination revealed two pulsatile, painless masses in the bilateral carotid regions. These masses were firm, fixed/nonmobile, and showed bruits on auscultation. There were no signs of overlying skin changes or tenderness.

**Fig. 1:**
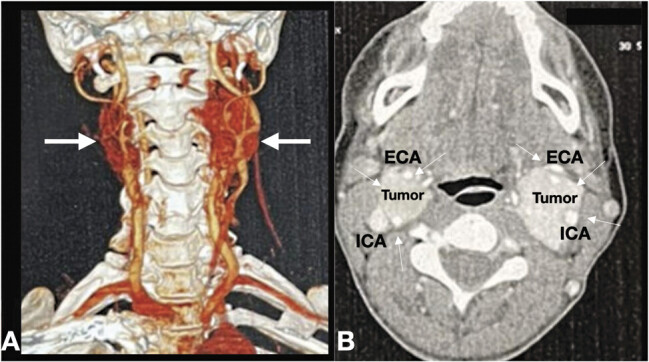
( **A** ) Three-dimensional CT angiography reformat image displaying bilateral carotid body tumors (white arrows). ( **B** ) Axial view showing bilateral solid masses with intense and slightly inhomogeneous enhancement at the level of the carotid bifurcation (white arrows). This lesion exhibits bright and rapid enhancement, illustrating a carotid body tumor that splayed the bifurcation of the internal carotid artery (ICA) and external carotid artery (ECA).

**Fig. 2:**
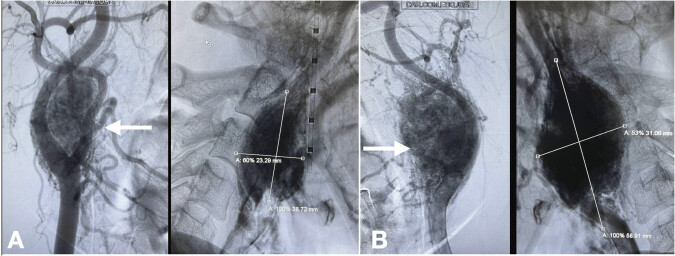
Angiography of the right ( **A** ) and left ( **B** ) internal carotid arteries demonstrating abnormal enhancement associated with the carotid body tumor. The cerebral blood flow study showed no alterations on either side.

A supra-aortic trunk angiotomography and angiography were conducted, revealing two heterogeneous nodular, hypervascular solid masses in each carotid space ( Fig. 1 ). The clinical and imaging findings suggested bilateral carotid body paragangliomas. Given the hypervascular nature of these tumors, a multidisciplinary approach was adopted. Surgical resection was denominated as the primary treatment. An angiography was necessary for surgical planning, providing a dynamic view of the tumor's vascular filling demonstrated a hypervascularized mass occupying and deforming the right-sided carotid bifurcation ( Fig. 2A ), as well as a formation in the region of the left-sided carotid bifurcation ( Fig. 2B ). The left-sided lesion was classified as a Shamblim type III mass, being larger than the right, which also fell under the category of Shamblim type III.

Preoperatively, optimizing blood pressure (BP) was crucial to mitigate the risks of autonomic instability and intraoperative bleeding. While alpha-blockade was not routinely required, antihypertensives were used as needed to maintain BP stability. In the absence of embolization, priority was given to hemoglobin optimization, fluid resuscitation planning, and blood crossmatching to manage the anticipated intraoperative blood loss. Additionally, a thorough neurological assessment was performed to establish a baseline for cranial nerve function and cerebral perfusion before carotid manipulation.

Postoperatively, BP fluctuations were carefully monitored, as bilateral CBT resection can impair baroreceptor reflexes, leading to hypertension, orthostatic hypotension, or labile BP responses. Short-acting IV antihypertensives were available for hypertensive episodes, while fluid resuscitation, vasopressors, and fludrocortisone were considered for persistent hypotension. Close neurological monitoring was essential for early detection of stroke, cranial nerve dysfunction, or airway compromise due to postoperative swelling or hematoma.

**Fig. 3:**
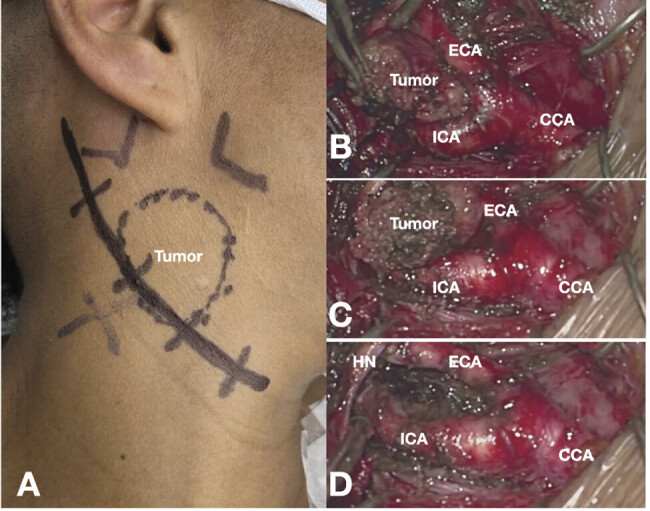
( **A** ) Photograph of the patient showing the preoperative planning for right-sided carotid body tumor surgery. A swelling in the upper lateral neck, corresponding to the tumor, can be observed. Intraoperative images ( **B–D** ) demonstrate a large tumor encapsulating the internal carotid artery (ICA) and external carotid artery (ECA), and adhering to and incorporating the hypoglossal nerve (HN). The final image ( **D** ) shows the outcome of the surgery, with complete resection of the tumor and preservation of the arteries.

Surgical treatment was prioritized on the right side. An oblique incision was made along the anterior border of the sternocleidomastoid muscle, extending proximally toward the mastoid process. The hypoglossal nerve was carefully isolated, and the vagus nerve, along with its pharyngeal and laryngeal branches, was identified. The tumor segment involving the artery was dissected, coagulated, and removed, exposing the arterial bifurcation. A subadventitial dissection technique was used to improve the exposure of the tumor, exposing and permitting the identification of the common carotid artery, internal carotid artery (ICA), and external carotid artery. Despite the increased risk of bleeding, preoperative embolization of the tumor was not performed, as it was believed that this would ease dissection and mobilization of the tumor. The main blood supply to the tumor was located at the bifurcation of the internal and external carotid arteries, as seen on dynamic angiographic analysis. Controlling these flowing arteries helped manage bleeding. The right-sided surgery resulted in a blood loss of 400 mL. Two months later, the tumor on the left side was resected, with a blood loss of 600 mL. Fragments were sent off confirming the diagnosis through histological analysis of the tissue fragments of both masses. There was no visible evidence of neurological deficit after the surgery ( Figs. 3 4 5 ).

**Fig. 4:**
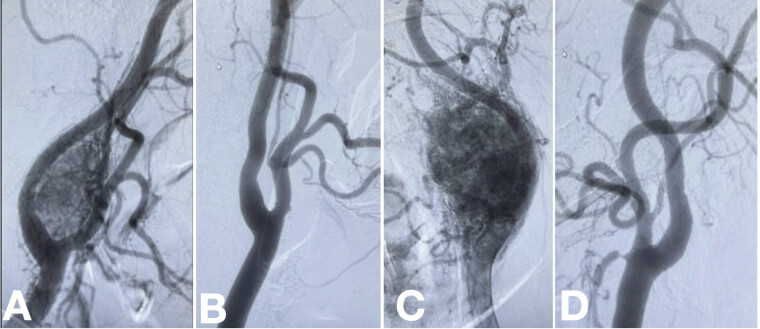
The images show preoperative angiography revealing a carotid body tumor that widens the bifurcation of the internal carotid artery (ICA) and external carotid artery (ECA) on the right side ( **A** ) and left side ( **C** ). Postoperative angiography of the right side ( **B** ) and left side ( **D** ) demonstrates complete resection of the tumor and preservation of the arteries.

**Fig. 5:**
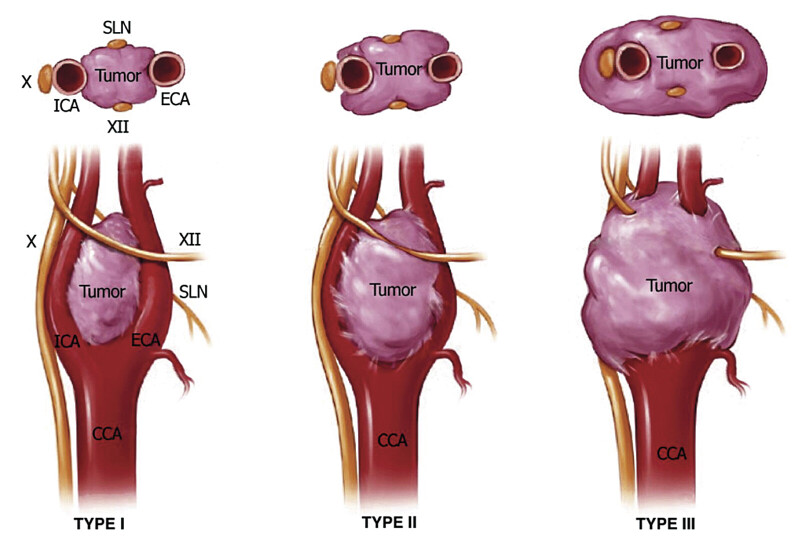
Shamblin classification. 5 Type I tumors are small lesions that do not splay the carotid bifurcation. Type II tumors are larger, significantly splay the carotid bifurcation, but do not circumferentially encase the carotid arteries. Type III tumors are large, encapsulate the internal or external carotid arteries, and often adhere to or incorporate the adjacent cranial nerves. CN, cranial nerve; ECA, external carotid artery; ICA, internal carotid artery; SLN, superior laryngeal nerve. (Adapted with permission from Hoang et al. 12 )

## Discussion

Paragangliomas of the carotid body are scarce, accounting for less than 0.5% of head and neck tumors. 3 Bilateral involvement is particularly unusual, presenting diagnostic and therapeutic challenges. These tumors originate from chemoreceptor cells involved in the process of blood oxygen level monitoring and are highly vascularized due to their paraganglial origin. 4

Clinical presentations of paragangliomas vary, with neck masses being one of the more common findings. Symptoms such as syncope, seen in our patient, are less common and may be related to baroreceptor dysfunction or compression of adjacent structures. Advanced imaging modalities, including CT angiography and MRI, are critical for diagnosis, allowing the detailed assessment of tumor size, vascularity, and anatomical relationships. 2 An angiography offers a dynamic view of the tumor's vascular supply and permits detailed visualization of any potential invasion into the affected artery. In the present case described, the arteries are displaced and dislocated by the tumor, but they remain free from invasion.

Surgical resection continues to be the main route of treatment for paragangliomas, though it poses challenges due to the proximity to critical vascular structures. Preoperative embolization can be considered an option to minimize intraoperative bleeding. 1 In cases of bilateral tumors, carefully planned surgical procedures may be needed to mitigate the risk of bilateral cranial nerve deficits. 4 In this case, although it was known that embolization could reduce blood loss, the surgical team opted to proceed with tumor resection without preoperative embolization. This decision was taken due to the fact that it would allow for better mobility of the tumor and the surrounding arteries during dissection. Additionally, the team successfully identified and coagulated the tumor's influx vessels, which provided improved control over bleeding.

Paragangliomas are classified according to a histological grading system that determines the tumor's aggressiveness, ranging from I to III. Grade III tumors are characterized by high aggressiveness, including necrosis, a high mitotic index, and the potential for local invasion and metastasis, making them challenging for surgical intervention. These characteristics increase the complexity and risk of surgery, often requiring a multifocal approach for appropriate management. 5

In this specific case, both paragangliomas were classified as grade III, indicating significant difficulty in surgery and a considerable risk of complications. Despite these challenges, the entire medical team adopted a well-thought-out surgical approach, resulting in successful resection and favorable recovery for the patient. This outcome exceeded expectations associated with the complexity of the tumors, demonstrating the success of the strategy employed and the effectiveness of the treatment. The clinical analysis highlights the importance of detailed evaluation and planning in difficult paraganglioma cases.

As mentioned previously for bilateral CBTs, the primary treatment is surgical resection, which has a high cure rate following complete resection of benign tumors. However, additionally, preoperative embolization is often used to reduce the tumor's vascularity and size, easing safer resection by decreasing blood loss. Another approach is carotid artery grafting and stenting to reduce blood supply to the tumor, which can also minimize its size and vascularity prior to surgery. In cases where surgical resection is not applicable or might result in significant neurological deficits, radiotherapy (RT) is employed as an alternative. RT can also be used for recurrent tumors or malignant CBTs. Additionally, chemotherapy is considered for malignant CBTs, although it is less effective compared to surgery. In managing bilateral cases, the strategy is typically to first operate on the smaller tumor to minimize the risk of baroreflex failure syndrome and other postoperative complications. If the initial surgery preserves nerve function, the contralateral tumor can be addressed; otherwise, RT may be recommended to avoid further deficits. This approach aims to balance tumor management with the minimization of surgical risks and complications. 6

Despite approximately 30% of bilateral CBTs following a familial autosomal dominant inheritance pattern, no other cases had been identified within the patient's family at the time. These tumors are typically associated with mutations in genes encoding subunits of the mitochondrial enzyme succinate dehydrogenase. Sporadic cases, such as this one, often harbor somatic mutations in genes involved in metabolism and DNA repair. However, a genetic workup had not been performed in this patient. 7

A variety of surgical management strategies exist for bilateral carotid body paragangliomas, each with distinct advantages and risks. Preoperative embolization, often used to reduce intraoperative blood loss, was not performed in this case due to concerns about ischemic complications such as embolic stroke, particularly given the tumor's proximity to the ICA and the complex collateral circulation in the head and neck. Embolization may also increase peritumoral inflammation and adhesions, potentially complicating surgical dissection rather than easing it. 8 9 Simultaneous bilateral resection was another potential approach but was avoided due to the high risk of baroreceptor failure and autonomic dysfunction, which could lead to severe BP instability, orthostatic hypotension, and an increased risk of cerebrovascular events. 10 Instead, a staged surgical approach was chosen, allowing the patient to adapt hemodynamically between procedures. RT, while an option for patients with unresectable or high-risk tumors, was not considered the optimal strategy in this case due to the patient's relatively young age, progressive symptoms, and the tumor's high-risk Shamblin III classification. Surgical resection offered definitive treatment, preventing further compression-related complications and tumor progression, whereas RT primarily stabilizes tumor growth and carries long-term risks such as vascular injury and cranial neuropathy. 11 Given these considerations, a staged surgical resection without embolization was determined to be the safest and most effective approach, considering tumor removal, hemodynamic stability, and neurological preservation.

## Conclusion

This case illustrates the clinical presentation, diagnostic investigation, and management of bilateral carotid body paragangliomas. Recognizing the characteristic features and employing proper imaging techniques are crucial for accurate diagnosis and effective treatment planning. The surgical techniques, as well as intimate knowledge of the neuroanatomy of the region, are essential for managing the complexities associated with bilateral paragangliomas to optimize patient outcomes.
